# Supplementary material for: Targeting branched N-glycans and fucosylation sensitizes ovarian tumors to immune checkpoint blockade
Source: Nat Commun. 2024 Apr 2;15:2853. doi: 10.1038/s41467-024-47069-y (PMC10987604; doi:10.1038/s41467-024-47069-y)
Supplement: Supplementary file 1 — Supplementary Information [file 41467_2024_47069_MOESM1_ESM.pdf]

Targeting Branched *N*-Glycans and Fucosylation Sensitizes Ovarian Tumors to Immune Checkpoint Blockade

Nie et al., Supplementary Information

Supplementary Table 1: List of mouse ovarian cancer cell lines used in the study.

Cell lines	Phenotypes	Genotypes	Source
ID8	HR-proficient	*Trp53*$^{+/+}$	PMID: 10753190
UPK10	HR-proficient	*Trp53*$^{-/-}$, *Kras*^{G12D}	PMID: 22351930
KPCA	HR-proficient	*Trp53*$^{-/-R172H}$, *Ccne1*^{OE}, *Akt2*^{OE}, *Kras*^{G12V}	PMID: 33158843
SPCA	HR-proficient	*Trp53*$^{-/-R172H}$, *Ccne1*^{OE}, *Akt2*^{OE}, *Smarca4*^{OE}	PMID: 33158843
BPCA	HR-proficient	*Trp53*$^{-/-R172H}$, *Ccne1*^{OE}, *Akt2*^{OE}, *Brd4*^{OE}	PMID: 33158843
PPNM	Non-classified	*Trp53*$^{-/-R172H}$, *Pten*$^{-/-}$, *Nf1*$^{-/-}$, *Myc*^{OE}	PMID: 33158843
BPPNM	HR-deficient	*Trp53*$^{-/-R172H}$, *Brca1*$^{-/-}$, *Pten*$^{-/-}$, *Nf1*$^{-/-}$, *Myc*^{OE}	PMID: 33158843
HGS2	HR-deficient	*Trp53*$^{-/-}$, *Brca2*$^{-/-}$, *Pten*$^{-/-}$	PMID: 31940494
ID8 (Brca2 KO)	HR-deficient	*Trp53*$^{-/-}$, *Brca2*$^{-/-}$	PMID: 27530326

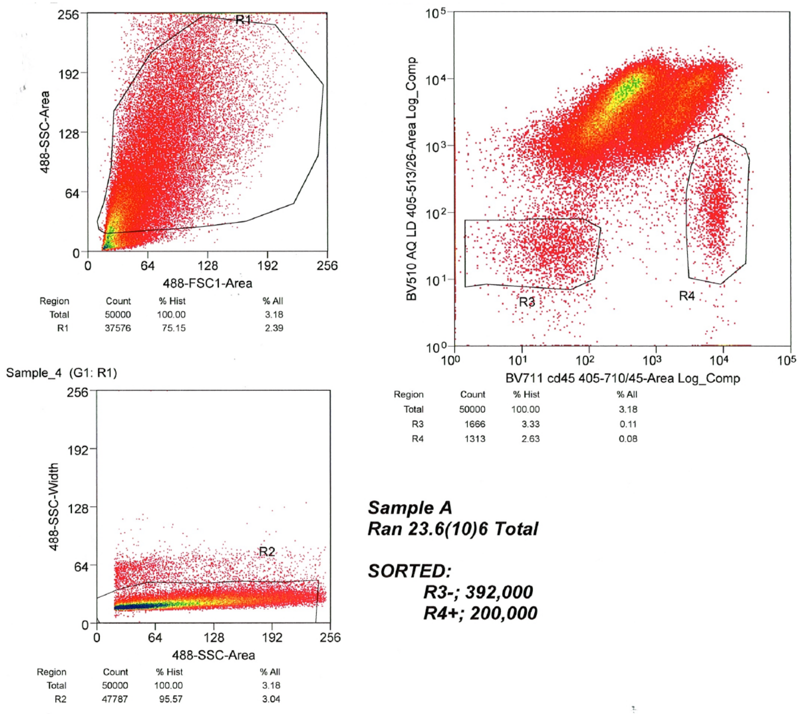

Supplementary Figure 1: An example of FACS sorting strategy for glycomic profile in Figure 1.

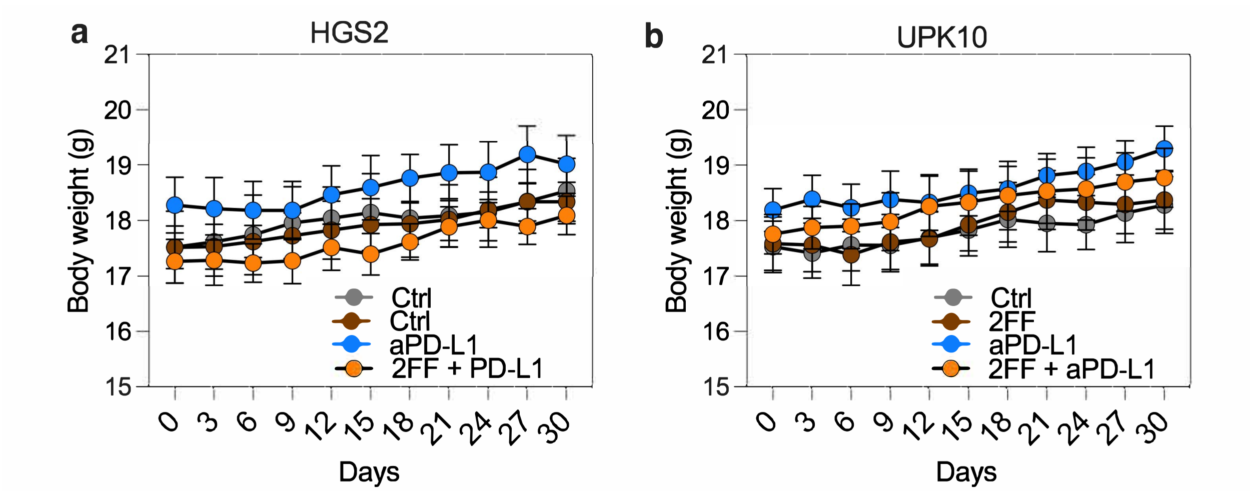

Supplementary Figure 2: 2FF and anti-PD-L1 combination treatment does not affect body weight of tumor-bearing mice.

Body weights of the mice in the indicated treatment groups after intrabursal injection in mice bearing HGS2 (**a**) and UPK10 (**b**) tumors. n=5 mice. Error bars represent Mean + SEM. Source data are provided as a Source Data file.

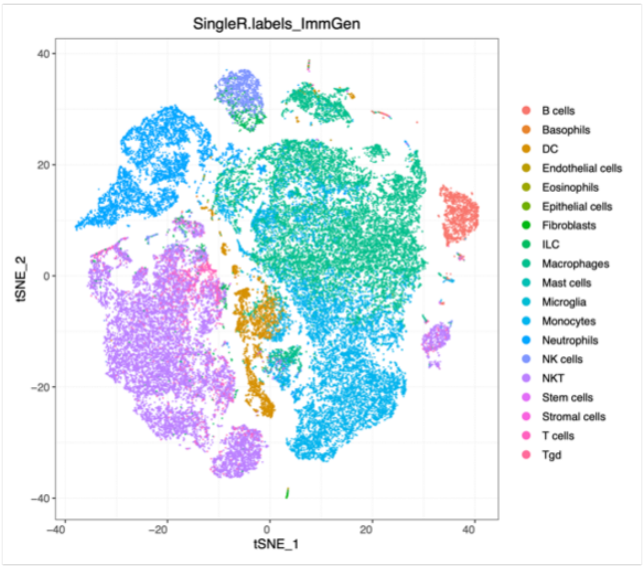

Supplementary Figure 3: Immune cell cluster analysis from the single-cell RNA sequencing experiments.
TSNE plot for unbiased clustering followed by prediction of cell types of cellular components of KPCA orthotopic tumors, where each color-coded cluster represents one immune cell type/state. Inset, relative composition of the clusters. Each point represents one cell that is colored by its cell type/state. n=6 mice pooled with one mouse from each of the experimental groups, namely: control, 2FF, 2DG, anti-PD-L1, 2FF/anti-PD-L1, and 2DG/anti-PD-L1.

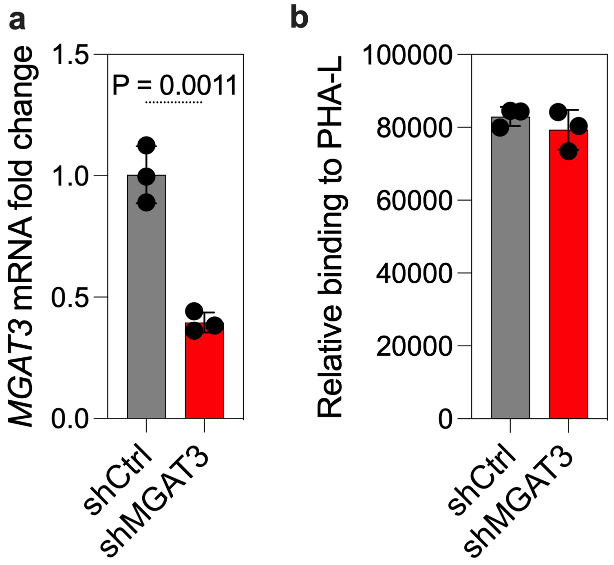

Supplementary Figure 4: MGAT3 expression does not affect branched *N*-glycans.
a-b, (a) HR-proficient cell line OVCAR3 expressing shMGAT3 and shControl were validated for MGAT3 knockdown by RT-qPCR, and (b) examined for the relative PHA-L binding to the cells by flow cytometry. Error bars represent Mean + SD. *P* values were calculated using two-tailed t-test. n = 3 biologically independent samples. Source data are provided as a Source Data file.

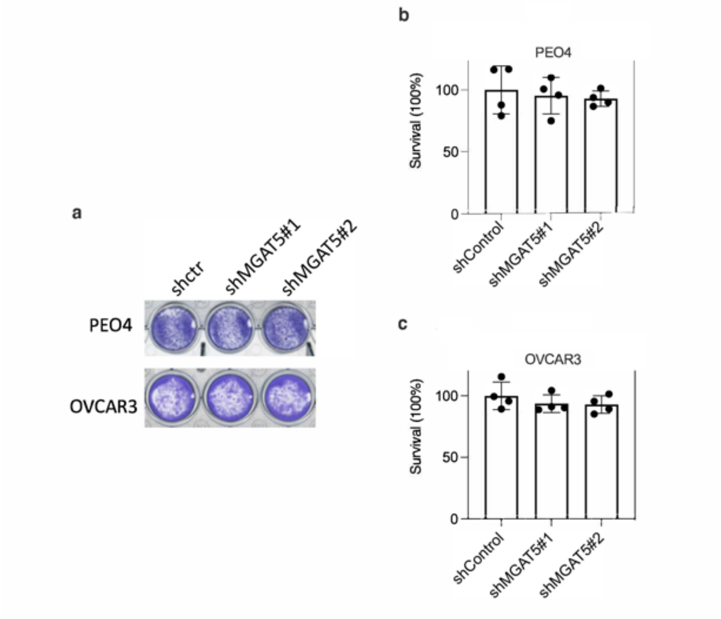

Supplementary Figure 5: Examination for proliferation of HR-proficient cell lines PEO4 and OVCAR3 expressing shMGAT5 and shControl.

3,000 cells were seeded into 24-well tissue culture plates. Cells were cultured in RPMI-1640 with 10% FBS. After 10 days, colonies were stained with 0.05% crystal violet (**a**), and integrated intensity was quantified for PEO4 (**b**) and OVCAR3 (**c**) cells. Error bars represent Mean + SD. n = 4 biologically independent samples. Source data are provided as a Source Data file.

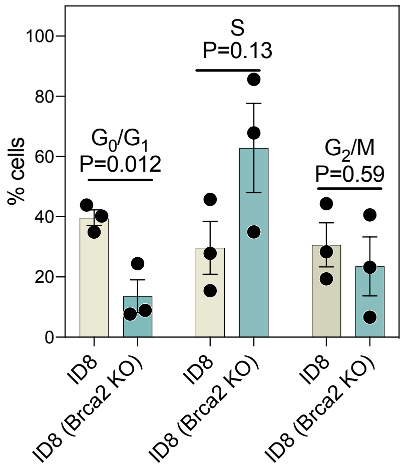

Supplementary Figure 6: *Brca2* knockout did not decrease percentages of cells in S phase of the cell cycle in ID8 cells.

Cell cycle distribution of the indicated cells was determined by FACS analysis. n = 3. Error bars represent Mean + SEM. *P* values were calculated using two-tailed t-test. n = 3 biologically independent samples. Source data are provided as a Source Data file.

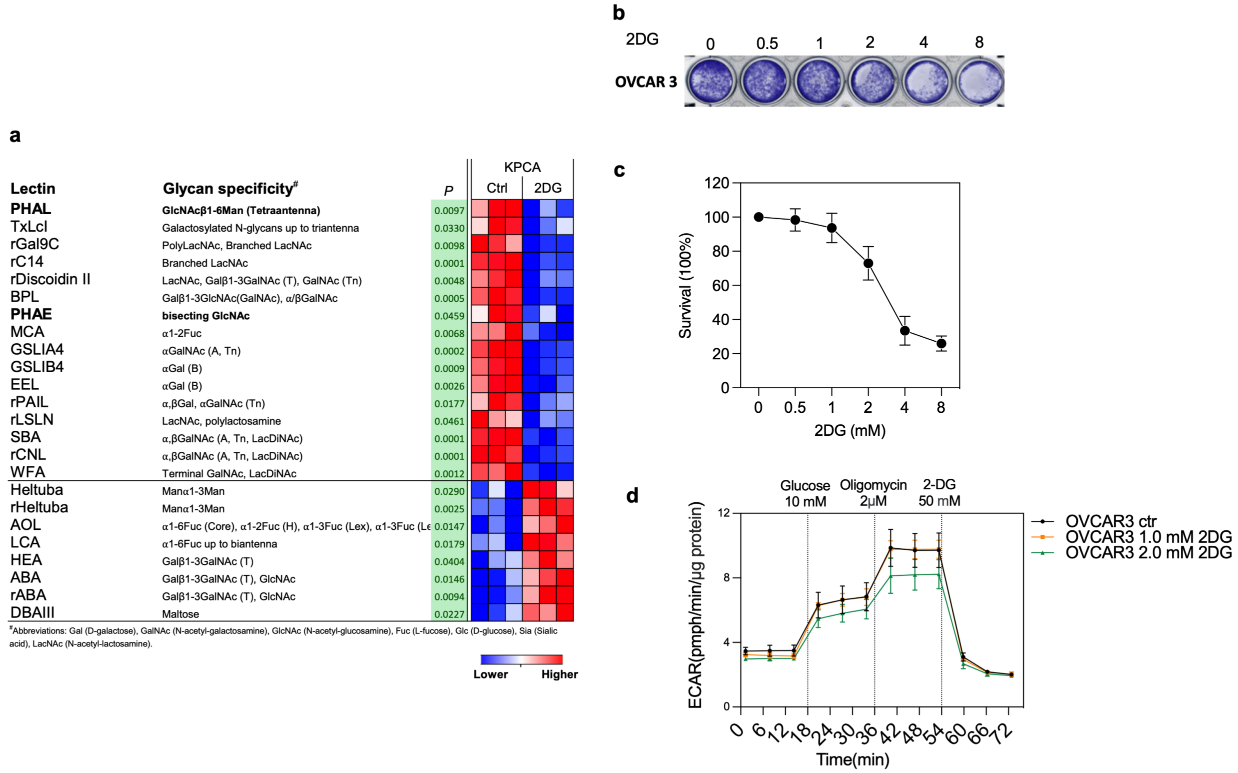

Supplementary Figure 7: 2DG effects on cancer cell glycomic profile, proliferation, and glycolysis.

a, Heatmap depicting the levels of specific glycan structures in untreated and 2DG treated group of KPCA cell line. *P* values were calculated using two-tailed t-test. n = 3 biologically independent samples. **b-c**, Examination for dose-response curves of OVCAR cells to 2DG. 3000 cells were first cultured in regular medium for 1 week, in the last 2 days, cells were cultured in the medium supplemented with the indicated concentration of 2DG. Afterwards, colonies were stained with 0.05% crystal violet (**b**) and the signal intensity was quantified using the National Institutes of Health ImageJ software (version 1.53a) (**c**). Each data point was the average of 4 independent measurements. Error bars represent mean with SD. **d,** The extracellular acidification rate (ECAR) of OVCAR3 cells treated with indicated concentration of 2DG. OVCAR3 cells were pretreated with appropriate doses of 2DG for 24 hours. Then, the cells (20,000 per well) were seeded and incubated with 2DG in an XF96 Cell Culture Microplate the day before running the assay. The media were exchanged to XF media 1 hour before the assay. Glucose, oligomycin, and 2-deoxyglucose (2-DG) were diluted into XF media and loaded into the cartridge to achieve final concentrations of 10, 2, and 50 mM, respectively. Each data point was the average of 4 independent measurements. *P* values were calculated using two-tailed t-test. Error bars represent mean with SD. Source data are provided as a Source Data file.

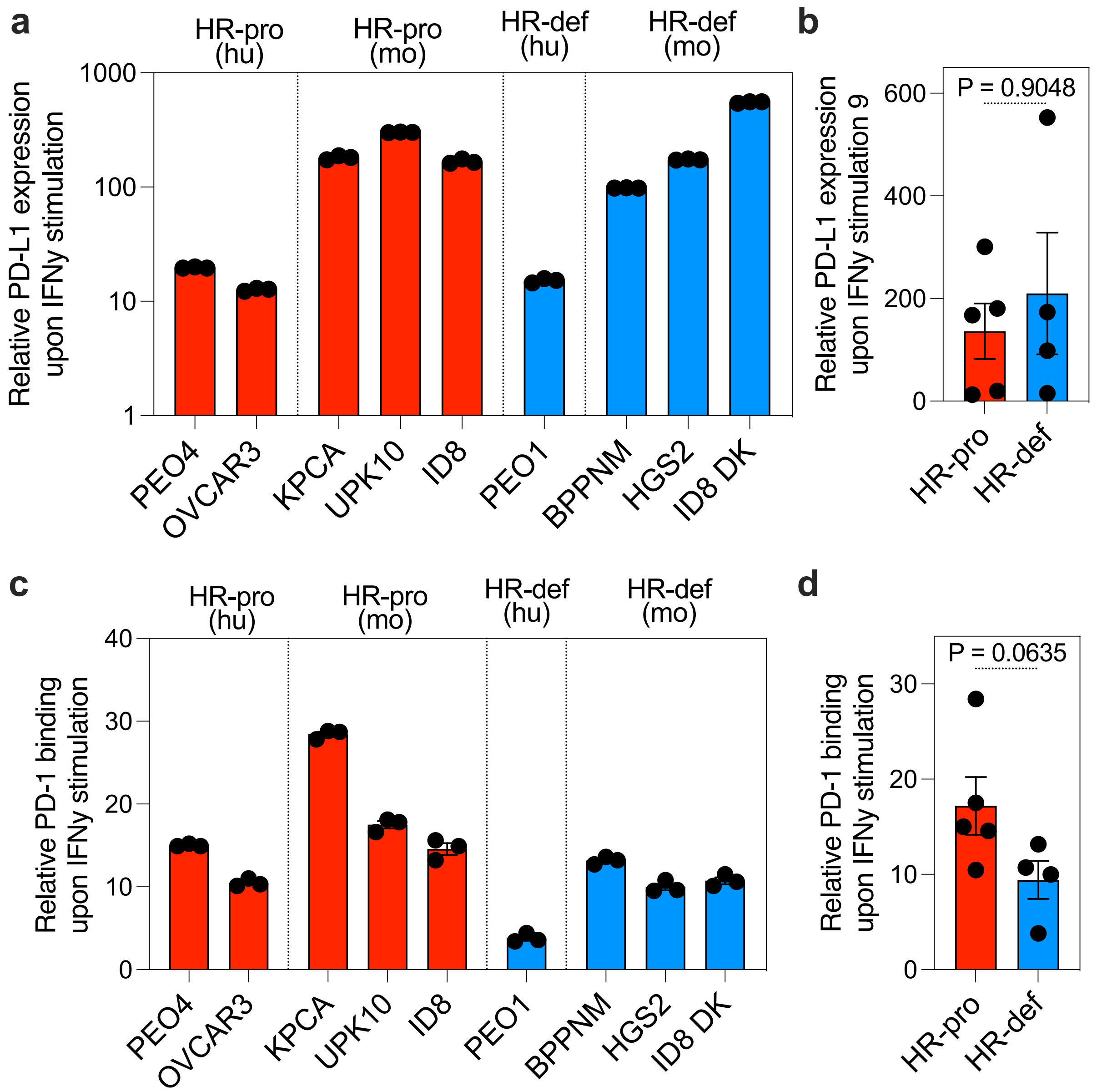

Supplementary Figure 8: Levels PD-L1 expression and PD-1 binding in several ovarian cancer cell lines by FACS analysis.

a, PD-L1 expression in several ovarian cancer cell lines. n = 3 biologically independent samples. **b,** Comparison of PD-L1 expression between HR-proficient and HR-deficient ovarian cancer cell lines. P value was generated using a non-parametric two-tailed t-test. Error bars represent the mean with SEM. n = 5 independent cell lines in HR-proficient group, n = 4 independent cell lines in HR-proficient group. **c,** Frequency of recombinant PD-1 binding to a series of ovarian cancer cell lines. n = 3 biologically independent samples. **d,** Comparison of recombinant PD-1 binding between HR-proficient and HR-deficient ovarian cancer cell lines. P value was generated using a non-parametric two-tailed t-test. Error bars represent the mean with SEM. n = 5 independent cell lines in HR-proficient group, n = 4 independent cell lines in HR-proficient group. Source data are provided as a Source Data file.

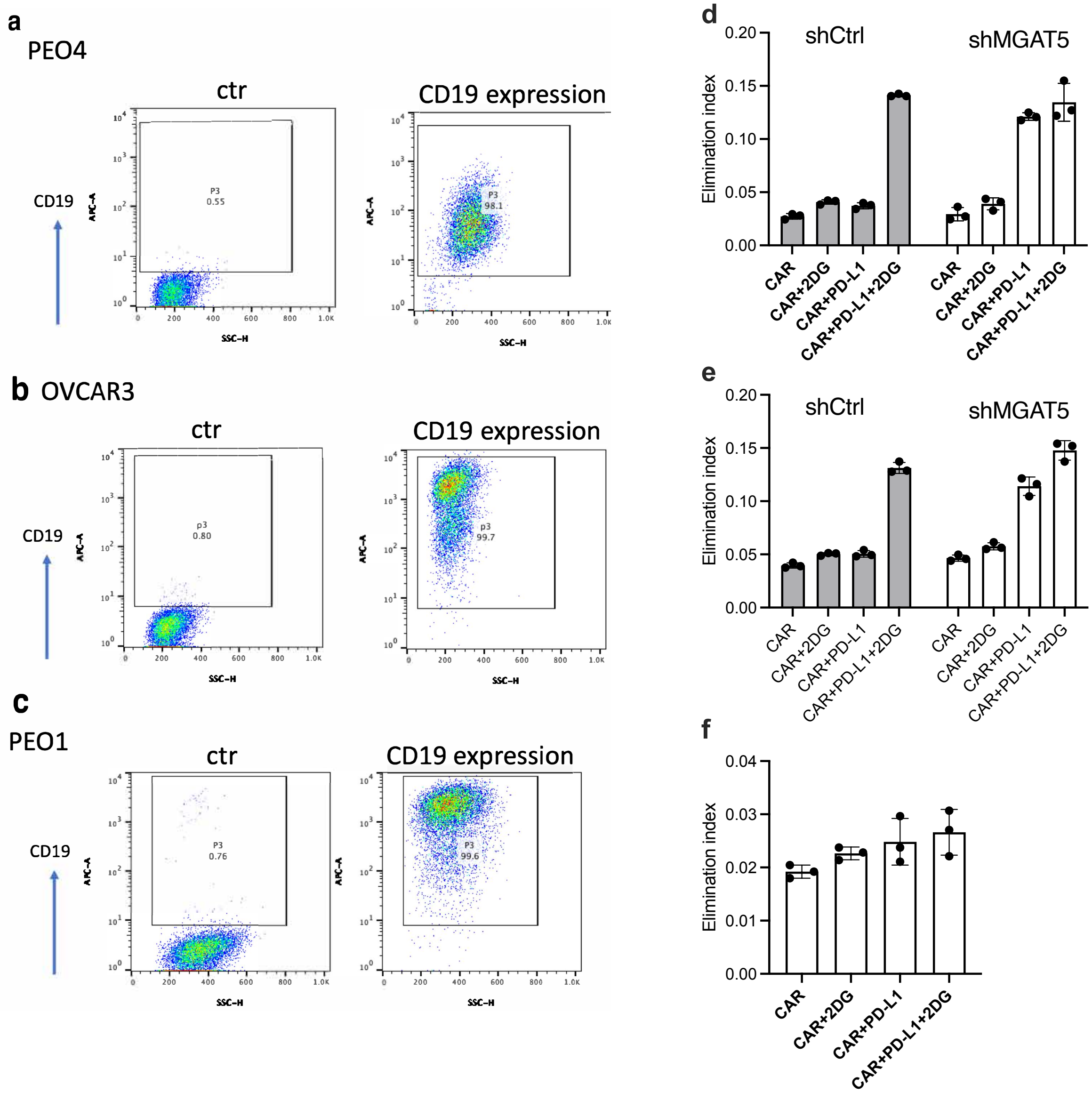

Supplementary Figure 9: Validation of CD19 ectopic expression in human ovarian cancer cells. a-c, Examination for CD19 expression on the surface of PEO4, OVCAR3 and PEO1 ectopically expressing CD19. Cells infected with vector (control) or CD19 lentivirus were stained by anti-human CD19 antibody (Biolegend, 302212) then analyzed by Flow cytometry. 3 biological repeats were performed for each sample. **d-f,** The base level of CAR T cells cytotoxicity against different ovarian cancer cells. **d,** Killing of HR-proficient PEO4 cells expressing CD19 was measured after coculture with anti-CD19 CAR T cells at the 1:6 E:T ratio (relative to Fig. 5e). **e,** Killing of HR-proficient OVCAR3 cells expressing CD19 was measured after coculture with anti-CD19 CAR T cells at the 1:6 E:T ratio (relative to Fig. 5f). **f,** Killing of HR-deficient PEO1 cells expressing CD19 was measured after coculture with anti-CD19 CAR T cells at the 1:6 E:T ratio (relative to Fig. 5g). Error bars represent the mean with SD. n = 3 biologically independent samples. Source data are provided as a Source Data file.

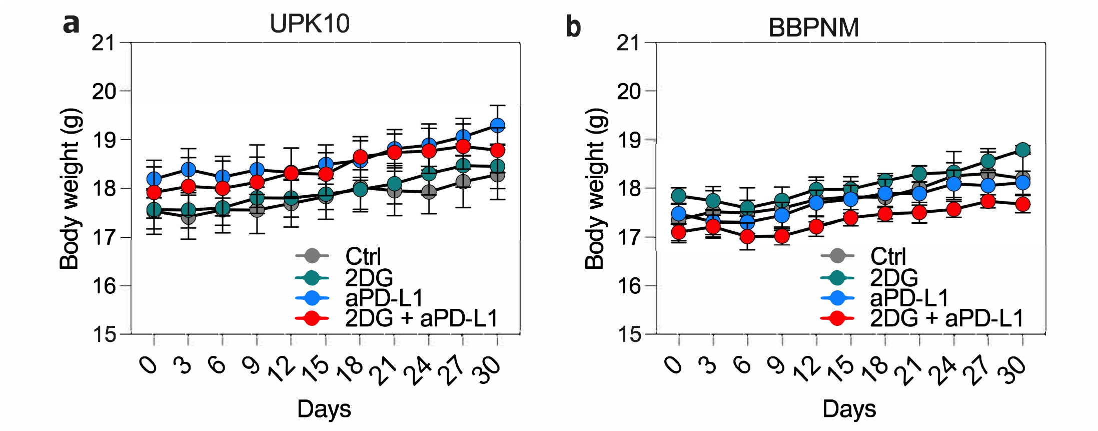

Supplementary Figure 10: 2DG and anti-PD-L1 combination treatment does not affect body weight of tumor-bearing mice.

Body weights of the mice in the indicated treatment groups after intrabursal injection in mice bearing UPK10 (**a**) or BPPNM (**b**) tumors. n = 5 mice per group with exception of n = 4 mice in BPPNM model. Error bars represent Mean + SEM. Source data are provided as a Source Data file.

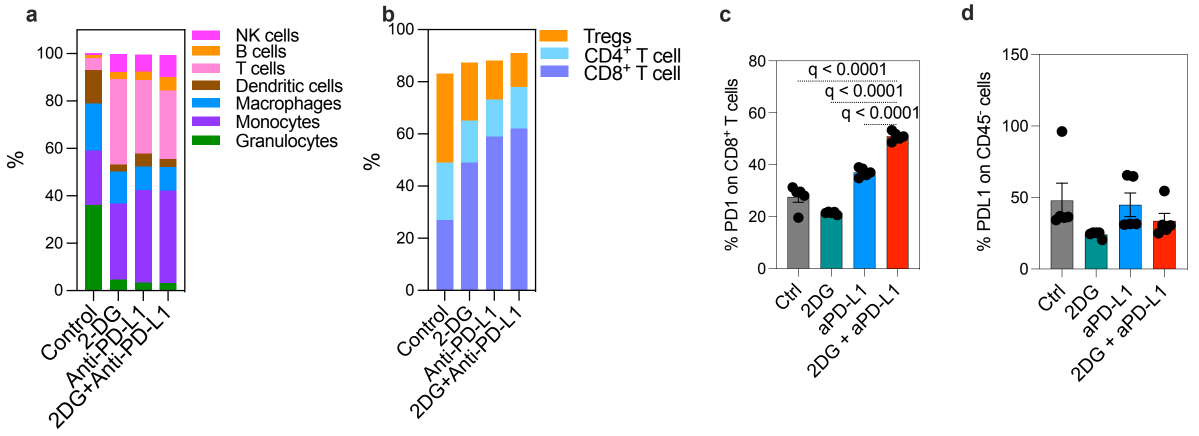

Supplementary Figure 11: Effects of 2DG + aPD-L1 treatment on immune profiles in vivo.

a, Selected Immune cell infiltration in each group of KPCA tumors based on single cell RNA-seq analysis. For single cell RNA-seq, n=1 mouse from each of the indicated groups. **b,** Selected T cell subsets infiltration in each group of KPCA tumors based on single cell RNA-seq analysis. For single cell RNA-seq, n=1 mouse from each of the indicated groups. **c**, FACS analysis of PD-1 expressed $CD8^+$ T cells in each group of KPCA tumors. Two-tailed *P* values were calculated by ANOVA corrected by the Benjamini, Krieger and Yekutieli method to generate q values. Error bars represent Mean + SEM. n = 5 mice. **d**, FACS analysis of PD-L1 expression in $CD45^-$ tumor cells in each group of KPCA tumors. Two-tailed *P* values were calculated by ANOVA corrected by the Benjamini, Krieger and Yekutieli method to generate q values. Error bars represent Mean + SEM. n = 5 mice. Source data are provided as a Source Data file.

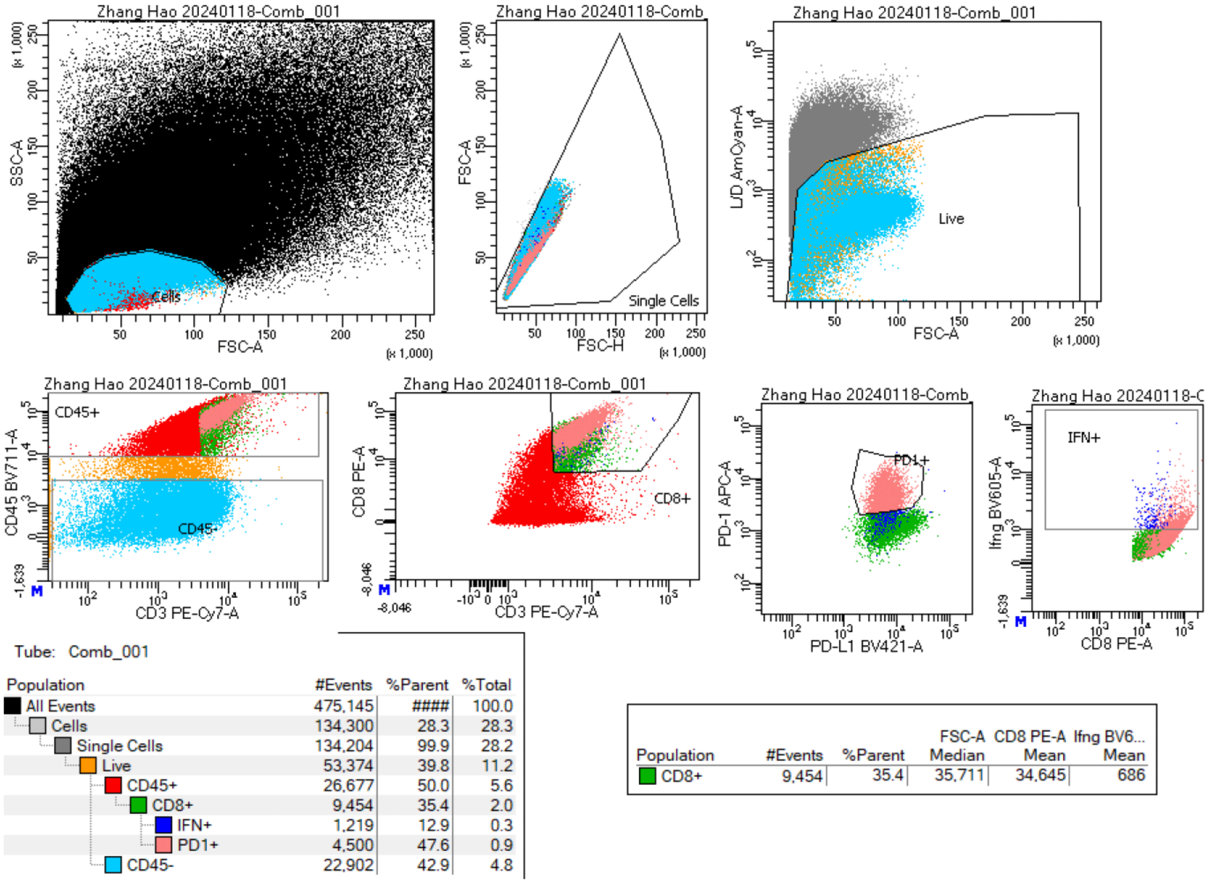

Tube: Comb_001

Population	#Events	%Parent	%Total
All Events	475,145	####	100.0
Cells	134,300	28.3	28.3
Single Cells	134,204	99.9	28.2
Live	53,374	39.8	11.2
CD45+	26,677	50.0	5.6
CD8+	9,454	35.4	2.0
IFN+	1,219	12.9	0.3
PD1+	4,500	47.6	0.9
CD45-	22,902	42.9	4.8

Population	#Events	%Parent	FSC-A Median	CD8 PE-A Mean	Ifng BV6... Mean
CD8+	9,454	35.4	35,711	34,645	686

Supplementary Figure 12: An example of FACS gating strategy used for Fig. 7c.
