# Supplementary material for: Targeting branched N-glycans and fucosylation sensitizes ovarian tumors to immune checkpoint blockade
Source: Nat Commun. 2024 Apr 2;15:2853. doi: 10.1038/s41467-024-47069-y (PMC10987604; doi:10.1038/s41467-024-47069-y)
Supplement: Supplementary file 3 — Reporting Summary [file 41467_2024_47069_MOESM3_ESM.pdf]

nature portfolio

Corresponding author(s): Rugang Zhang, Ph.D.

Last updated by author(s): Mar 7, 2024

Reporting Summary

Nature Portfolio wishes to improve the reproducibility of the work that we publish. This form provides structure for consistency and transparency in reporting. For further information on Nature Portfolio policies, see our Editorial Policies and the Editorial Policy Checklist.

Statistics

For all statistical analyses, confirm that the following items are present in the figure legend, table legend, main text, or Methods section.

n/a	Confirmed	
☐	☒	The exact sample size (*n*) for each experimental group/condition, given as a discrete number and unit of measurement
☐	☒	A statement on whether measurements were taken from distinct samples or whether the same sample was measured repeatedly
☐	☒	The statistical test(s) used AND whether they are one- or two-sided<br>*Only common tests should be described solely by name; describe more complex techniques in the Methods section.*
☒	☐	A description of all covariates tested
☒	☐	A description of any assumptions or corrections, such as tests of normality and adjustment for multiple comparisons
☐	☒	A full description of the statistical parameters including central tendency (e.g. means) or other basic estimates (e.g. regression coefficient) AND variation (e.g. standard deviation) or associated estimates of uncertainty (e.g. confidence intervals)
☐	☒	For null hypothesis testing, the test statistic (e.g. *F*, *t*, *r*) with confidence intervals, effect sizes, degrees of freedom and *P* value noted<br>*Give P values as exact values whenever suitable.*
☒	☐	For Bayesian analysis, information on the choice of priors and Markov chain Monte Carlo settings
☒	☐	For hierarchical and complex designs, identification of the appropriate level for tests and full reporting of outcomes
☐	☒	Estimates of effect sizes (e.g. Cohen's *d*, Pearson's *r*), indicating how they were calculated

Our web collection on statistics for biologists contains articles on many of the points above.

Software and code

Policy information about availability of computer code

Data collection	Flow cytometry data were acquired using BD FACSDiva Version 8.0<br>Images were analyzed using Mac OS X version of NIH Image J software
Data analysis	Prism 7 for Mac was used for calculating p values,<br>Flow cytometry results were analyzed using FlowJo v10.0.7 software<br>The intensity of immunoblotting was quantified by Mac OS X version of NIH Image J software<br>The intensity of colony formation was quantified by Mac OS X version of NIH Image J software<br>Glycomic profile data were analyzed by R package limma v.3.46.0<br>For scRNA-seq, the R package SingleR was used to determine cell types of the clusters using the ImmGen dataset as a reference for cell-specific gene signatures.

For manuscripts utilizing custom algorithms or software that are central to the research but not yet described in published literature, software must be made available to editors and reviewers. We strongly encourage code deposition in a community repository (e.g. GitHub). See the Nature Portfolio guidelines for submitting code & software for further information.

Data

Policy information about availability of data

All manuscripts must include a data availability statement. This statement should provide the following information, where applicable:

- Accession codes, unique identifiers, or web links for publicly available datasets
- A description of any restrictions on data availability
- For clinical datasets or third party data, please ensure that the statement adheres to our policy

The scRNA-seq data generated in this study have been deposited in the NCBI database under accession code GSE244012. The online ChIP-seq data that we reanalyzed here are available in the Gene Expression Omnibus (GEO) database under accession codes GSE170476 and GSM935552. Mutation status, gene expression and survival data of HGSOCs were downloaded from MDACC TCGA data portal (https://bioinformatics.mdanderson.org/MQA/) and cBioPortal (https://www.cbioportal.org/). Mutation status and gene expression for cell lines were downloaded from DepMap portal (https://depmap.org/portal/download, dataset DepMap Public 22Q2). Source data are provided with this paper.

Research involving human participants, their data, or biological material

Policy information about studies with human participants or human data. See also policy information about sex, gender (identity/presentation), and sexual orientation and race, ethnicity and racism.

Reporting on sex and gender	N/A
Reporting on race, ethnicity, or other socially relevant groupings	N/A
Population characteristics	N/A
Recruitment	N/A
Ethics oversight	N/A

Note that full information on the approval of the study protocol must also be provided in the manuscript.

Field-specific reporting

Please select the one below that is the best fit for your research. If you are not sure, read the appropriate sections before making your selection.

[x] Life sciences [ ] Behavioural & social sciences [ ] Ecological, evolutionary & environmental sciences

For a reference copy of the document with all sections, see nature.com/documents/nr-reporting-summary-flat.pdf

Life sciences study design

All studies must disclose on these points even when the disclosure is negative.

Sample size	No sample size was pre-determined. Sample size and number of independent experiments are stated in the figure legend or in the Methods or Results section. Three or more independent results were used to perform statistical analyses. Studies involving independent cohorts of mice were typically performed once, with several exceptions stated in the figure legends. No specific statistical tests were applied to determine the sample size, the size was established according to our previous experience with the models used.
Data exclusions	There was no exclusion from the experiments.
Replication	All experiments in the manuscript were performed at least twice independently. Each experiment in vitro contained 3 independent replicates per sample. Each experiment in vivo was performed with n=4-5 mice/group. All attempts at replication were successful. Exact numbers of biologically independent experimental repetition are stated in the manuscript.
Randomization	Experiments are all randomized
Blinding	Investigators were blinded during data collection and analysis.

Reporting for specific materials, systems and methods

We require information from authors about some types of materials, experimental systems and methods used in many studies. Here, indicate whether each material, system or method listed is relevant to your study. If you are not sure if a list item applies to your research, read the appropriate section before selecting a response.

Materials & experimental systems

n/a	Involved in the study	
☐	☒	Antibodies
☐	☒	Eukaryotic cell lines
☒	☐	Palaeontology and archaeology
☐	☒	Animals and other organisms
☒	☐	Clinical data
☒	☐	Dual use research of concern
☒	☐	Plants

Methods

n/a	Involved in the study	
☒	☐	ChIP-seq
☐	☒	Flow cytometry
☒	☐	MRI-based neuroimaging

Antibodies

Antibodies used

For immunoblotting:
For primary antibodies: anti-β-actin (1:1000; Cell Signaling, Cat#: 4970, RRID:AB_2223172), anti-BRCA2 (1:500; Cell Signaling, Cat#: 10741, RRID:AB_2797730), anti-BRCA1 C-terminus (1:200; Santa Cruz, Cat#: sc-6954, RRID: AB_626761), anti-BRCA1 N-terminus (1 µg/mL, R&D Systems, Cat#: AF2210, RRID:AB_2067618), anti-MGAT5 for mouse (1:500; Thermo Fisher, Cat#: PA5-87988, RRID:AB_2804566), anti-MGAT5 for human (1:500; R&D Systems, Cat#: MAB5469, RRID:AB_10972310).
For secondary antibodies: Anti-mouse IgG, HRP-linked Antibody (1:2000; Cell Signaling, Cat#: 7076, RRID:AB_330924), Anti-rabbit IgG, HRP-linked Antibody (Cat#: 7074, RRID:AB_2099233), anti-Goat IgG (H+L) Secondary Antibody, HRP (1:5000; Thermo Fisher, Cat#: 31402, RRID:AB_228395)

For regular FACS analysis: APC anti-mouse CD274 (B7-H1, PD-L1) antibody Biolegend, 124311, RRID:AB_10612935) was used at a 1:100 dilution. PE anti-human CD274 (B7-H1, PD-L1) antibody (Biolegend, 329706, RRID:AB_940368) was used at a 1:20 dilution. APC anti-human CD19 antibody (Biolegend, 302212, RRID:AB_314242) was used at a 1:20 dilution.

For Chip-qPCR: anti-BRCA1 antibody (Santa Cruz, sc-6954, RRID:AB_626761, 5 µg/IP) and anti-BRCA2 antibody (Cell Signaling, 10741, RRID:AB_2797730, 10 µL/IP)

For immune cell FACS analysis: Hamster anti-mouse CD3ε (1:100; Biolegend, Cat#: 100320, RRID:AB_312684, 1:200), rat anti-mouse CD45 (1:100; Biolegend, Cat#: 103147, RRID:AB_2564383, 1:200), rat anti-mouse CD8a (1:100; Biolegend, Cat#: 100707, RRID:AB_312747, 1:200), rat anti-mouse PD-1 (1:100; Biolegend, Cat#: 135209, RRID:AB_2251944, 1:200), rat anti-mouse PD-L1 (1:100; Biolegend, Cat#: 124315, RRID:AB_10897097, 1:200) and rat anti-mouse IFN-γ antibody (1:100; Biolegend, Cat#: 505840, RRID:AB_2734493, 1:200).

Validation

Information of each antibody is available on the manufacturer's website.

For immunoblotting, all the antibodies were validated by the manufacturers, anti-MGAT5 for human (1:500; R&D Systems, Cat#: MAB5469, RRID:AB_10972310), anti-BRCA2 (1:500; Cell Signaling, Cat#: 10741, RRID:AB_2797730) anti-BRCA1 C-terminus (1:200; Santa Cruz, Cat#: sc-6954, RRID: AB_626761), anti-BRCA1 N-terminus (1 µg/mL, R&D Systems, Cat#: AF2210, RRID:AB_2067618), Anti-mouse IgG, HRP-linked Antibody (1:2000; Cell Signaling, Cat#: 7076, RRID:AB_330924), Anti-rabbit IgG, HRP-linked Antibody (Cat#: 7074, RRID:AB_2099233), anti-Goat IgG (H+L) Secondary Antibody, HRP (1:5000; Thermo Fisher, Cat#: 31402, RRID:AB_228395) used in this manuscript for WB analysis have accompanying knockdown data.

For Chip-qPCR: anti-BRCA1 (Santa Cruz, sc-6954, RRID:AB_626761) and anti-BRCA2 antibody (Cell Signaling, 10741, RRID:AB_2797730) are validated in HR-proficient human cancer cell lines PEO4 and OVCAR3.

For flow: anti-mouse CD274 (B7-H1, PD-L1) antibody (Biolegend, 124311, RRID:AB_10612935) was by the manufacturer in C57/B6 mouse splenocytes, anti-human CD274 (B7-H1, PD-L1) antibody (Biolegend, 329706, RRID:AB_940368) was validated by the manufacturer in human peripheral blood lymphocytes, anti-human CD19 antibody was validated by the manufacturer in human peripheral blood lymphocytes.
anti-mouse CD3ε (Biolegend, Cat#: 100320, RRID:AB_312684) was validated by the manufacturer in C57BL/6 mouse splenocytes, anti-mouse CD45 (Biolegend, Cat#: 103147, RRID:AB_2564383) was validated by the manufacturer in C57BL/6 mouse splenocytes, anti-mouse CD8a (Biolegend, Cat#: 100707, RRID:AB_312747) was validated by the manufacturer in C57BL/6 mouse splenocytes, anti-mouse PD-1 (Biolegend, Cat#: 135209, RRID:AB_2251944) was validated by the manufacturer in C57BL/6 mouse splenocytes, anti-mouse PD-L1 (Biolegend, Cat#: 124315, RRID:AB_10897097) was validated by the manufacturer in C57BL/6 mouse splenocytes, anti-mouse IFN-γ antibody (Biolegend, Cat#: 505840, RRID:AB_2734493) was validated by the manufacturer in C57BL/6 mouse splenocytes.

Eukaryotic cell lines

Policy information about cell lines and Sex and Gender in Research

Cell line source(s)

HEK 293T and OVCAR3 were purchased from ATCC.
PEO1 cell line was obtained from Dr. Thomas Hamilton (Fox Chase Cancer Center).

	PEO4 was purchased from Sigma (Cat#: 10032309)<br>KPCA, BPCA, SPCA, PPNM, BPPNM, HGS2, UPK10, ID8 and ID8 (Trp53 ko, Brca2 ko) were obtained as previously published.
Authentication	Cell lines were re-authenticated by The Wistar Institute's Genomics Facility using short tandem repeat profiling using AmpFLSTR Identifiler PCR Amplification kit (Life Technologies).
Mycoplasma contamination	Regular Mycoplasma testing was performed using LookOut Mycoplasma PCR detection (Sigma). All cell lines applied in this study were tested negative for Mycoplasma.
Commonly misidentified lines<br>(See ICLAC register)	No commonly misidentified cell lines used.

Animals and other research organisms

Policy information about studies involving animals; ARRIVE guidelines recommended for reporting animal research, and Sex and Gender in Research

Laboratory animals	6-8 week female NSG mice were purchased from Wistar Institute Animal Facility. 6-week female C57BL/6 mice were purchased from Charles River Laboratories.<br>Mice were maintained at 22–23 °C with 40–60% humidity and a 12-h light–12-h dark cycle.
Wild animals	No wild animals were used in the study
Reporting on sex	Only female mice were used in this study
Field-collected samples	No field-collected samples were used in this study
Ethics oversight	Animal protocols were approved by the Institutional Animal Care and Use Committee (IACUC) of The Wistar Institute and the University of Texas MD Anderson Cancer Center

Note that full information on the approval of the study protocol must also be provided in the manuscript.

Flow Cytometry

Plots

Confirm that:

- [x] The axis labels state the marker and fluorochrome used (e.g. CD4-FITC).
- [x] The axis scales are clearly visible. Include numbers along axes only for bottom left plot of group (a 'group' is an analysis of identical markers).
- [x] All plots are contour plots with outliers or pseudocolor plots.
- [x] A numerical value for number of cells or percentage (with statistics) is provided.

Methodology

Sample preparation	For regular FACS analysis, Cells were washed with PBS containing 0.5% (w/v) BSA (PBS/BSA). All staining was carried out in this buffer. For directly labeled antibodies and lectins, cells were then washed two times with cold PBS/BSA buffer, resuspended in this buffer, and analyzed by flow cytometry.<br>For tumor immune cell FACS analysis, Tumors were chopped and digested with Mouse Dissociation Kit (Miltenyi Biotec, 130-096-730). Single cells were then harvested with 70 mm strainer and used for staining. Live/dead cells were discriminated by viability staining Kit (Thermo Fisher, Cat#: L34965)
Instrument	LSRII-14 flow cytometer, LSRII-18 flow cytometer and BD LSRFortessa™ Cell Analyzer
Software	Data were acquired using BD FACSDiva Version 8.0 and analyzed using FlowJo v10.0.7 software
Cell population abundance	Cell sorting strategy for glycomic profile is shown in Supplementary Fig. 1.
Gating strategy	Cell debris were removed using FSC-A and SSC-A. Doublet were removed from total population using FSC-A and FSC-H. Positive population were gated based on comparing on unstained samples and single stained sample.<br>Gating strategy for immune cell analysis is shown in Supplementary Fig. 12.

- [x] Tick this box to confirm that a figure exemplifying the gating strategy is provided in the Supplementary Information.
